# Supplementary material for: CA2 neuronal activity controls hippocampal low gamma and ripple oscillations
Source: eLife. 2018 Nov 2;7:e38052. doi: 10.7554/eLife.38052 (PMC6251629; doi:10.7554/eLife.38052)
Supplement: Transparent reporting form [file elife-38052-transrepform.docx]

***eLife’s* transparent reporting form**

We encourage authors to provide detailed information *within their submission* to facilitate the interpretation and replication of experiments. Authors can upload supporting documentation to indicate the use of appropriate reporting guidelines for health-related research (see [EQUATOR Network](http://www.equator-network.org/%20)), life science research (see the [BioSharing Information Resource](https://biosharing.org/)), or the [ARRIVE guidelines](http://www.plosbiology.org/article/info:doi/10.1371/journal.pbio.1000412) for reporting work involving animal research. Where applicable, authors should refer to any relevant reporting standards documents in this form.

If you have any questions, please consult our Journal Policies and/or contact us: [editorial@elifesciences.org](mailto:editorial@elifesciences.org).

**Sample-size estimation**

- You should state whether an appropriate sample size was computed when the study was being designed
- You should state the statistical method of sample size computation and any required assumptions
- If no explicit power analysis was used, you should describe how you decided what sample (replicate) size (number) to use

Please outline where this information can be found within the submission (e.g., sections or figure legends), or explain why this information doesn’t apply to your submission:

Page 23, final paragraph 1 (Methods section): No statistical tests were used to determine sample sizes a priori, but sample sizes for histological and electrophysiological studies were similar to those used in the field.

**Replicates**

- You should report how often each experiment was performed
- You should include a definition of biological versus technical replication
- The data obtained should be provided and sufficient information should be provided to indicate the number of independent biological and/or technical replicates
- If you encountered any outliers, you should describe how these were handled
- Criteria for exclusion/inclusion of data should be clearly stated
- High-throughput sequence data should be uploaded before submission, with a private link for reviewers provided (these are available from both GEO and ArrayExpress)

Please outline where this information can be found within the submission (e.g., sections or figure legends), or explain why this information doesn’t apply to your submission:

The number of biological replicates for every measurement and statistical analysis is listed with the statistic throughout the Results section and in corresponding figure legends.

The following paragraph is included in the Methods section on page 17:

Results Reporting and Data Availability

For each experiment presented within the Results section and in figures, the number of replicates is presented as “N” when indicating the number of animals that were used for the experiment or as “n” when referring to the number of neurons used for the experiment. Statistical tests used for each experiment are presented in the text. Statistical significance was based on a p-value of 0.05. All error bars in graphs represent standard error of the mean. The data used to generate bar graphs in figures are listed in Supplemental Table 1.

**Statistical reporting**

- Statistical analysis methods should be described and justified
- Raw data should be presented in figures whenever informative to do so (typically when N per group is less than 10)
- For each experiment, you should identify the statistical tests used, exact values of N, definitions of center, methods of multiple test correction, and dispersion and precision measures (e.g., mean, median, SD, SEM, confidence intervals; and, for the major substantive results, a measure of effect size (e.g., Pearson's r, Cohen's d)
- Report exact p-values wherever possible alongside the summary statistics and 95% confidence intervals. These should be reported for all key questions and not only when the p-value is less than 0.05.

Please outline where this information can be found within the submission (e.g., sections or figure legends), or explain why this information doesn’t apply to your submission:

The number of biological replicates for every measurement and statistical analysis used for comparison is listed throughout the Results section and in corresponding figure legends. Exact p values are presented for all statistical tests, unless a p value of <0.0001 was obtaining, in which case Prism statistical software does not provide an exact p value.

The following information is provided in the Methods section on page 16, paragraph 2:

Power and coherence values measured for each treatment were compared using appropriate statistical tests, listed in text, after data were checked for normal distributions and equal variance.

The following information is provided in the Methods section on page 17, paragraph 1:

Ripple event frequency and ripple amplitude were measured and appropriate statistical tests were applied, as listed in the text, after data were checked for normal distributions and equal variance.

The following paragraph is included in the Methods section on page 17:

Results Reporting and Data Availability

For each experiment presented within the Results section and in figures, the number of replicates is presented as “N” when indicating the number of animals that were used for the experiment or as “n” when referring to the number of neurons used for the experiment. Statistical tests used for each experiment are presented in the text. Statistical significance was based on a p-value of 0.05. All error bars in graphs represent standard error of the mean. The data used to generate bar graphs in figures are listed in Supplemental Table 1.

(For large datasets, or papers with a very large number of statistical tests, you may upload a single table file with tests, Ns, etc., with reference to sections in the manuscript.)

**Group allocation**

- Indicate how samples were allocated into experimental groups (in the case of clinical studies, please specify allocation to treatment method); if randomization was used, please also state if restricted randomization was applied
- Indicate if masking was used during group allocation, data collection and/or data analysis

Please outline where this information can be found within the submission (e.g., sections or figure legends), or explain why this information doesn’t apply to your submission:

For group allocation, the following information appears in the Methods section on page 12, paragraph 1:

For electrophysiology studies, *Amigo2*-icreERT2+ and *Amigo2*-icreERT2- animals were randomly selected from litters. For randomization, animals were housed with same-sex littermates following weaning but before genotyping. Genotype information was unknown at the time of randomly selecting a mouse from the cage for AAV infusion.

For masking, the following information appears in the Methods section on page 16, paragraph 1:

The experimenter was blind to the genotype of animals at the time of data analysis.

**Additional data files (“source data”)**

- We encourage you to upload relevant additional data files, such as numerical data that are represented as a graph in a figure, or as a summary table
- Where provided, these should be in the most useful format, and they can be uploaded as “Source data” files linked to a main figure or table
- Include model definition files including the full list of parameters used
- Include code used for data analysis (e.g., R, MatLab)
- Avoid stating that data files are “available upon request”

Please indicate the figures or tables for which source data files have been provided:

The data used to generate bar graphs in main text figures (Figures 2-6) are listed in Supplemental Table 1.
